# Are mosquito species present in Spain competent for Oropouche virus?

**DOI:** 10.1186/s13071-026-07472-4

**Published:** 2026-06-05

**Authors:** Rafael Gutiérrez-López, Nuria Labiod, Marcos López-de-Felipe, Patricia Sánchez-Mora, Sarah Delacour-Estrella, Alexandra Martín-Ramírez, Arantxa Potente, Eva Pérez-Martínez, Ricardo Molina, Maripaz Sánchez-Seco, Maribel Jiménez, Ana Vázquez, Inés Martín-Martín

**Affiliations:** 1 Laboratory of Arboviruses, National Center for Microbiology, Instituto de Salud Carlos III, 28220 Majadahonda, Madrid Spain; 2 Consorcio de Investigación Biomédica en Red de Enfermedades Infecciosas (CIBERINFEC), Instituto de Salud Carlos III, Madrid, Spain; 3 Laboratory of Medical Entomology, National Center for Microbiology, Instituto de Salud Carlos III, 28220 Majadahonda, Madrid Spain; 4 Animal Health Department, The AgriFood Institute of Aragon (IA2), School of Veterinary Medicine, University of Zaragoza, 50013 Zaragoza, Spain; 5 Malaria and Emerging Parasitic Diseases Laboratory, National Center for Microbiology, Instituto de Salud Carlos III, Majadahonda, Madrid Spain; 6 Consorcio de Investigación Biomédica en Red de Epidemiología y Salud Pública (CIBERESP), Instituto de Salud Carlos III, Madrid, Spain

**Keywords:** Arboviruses, *Aedes albopictus*, *Culex pipiens*, *Aedes aegypti*, *Orthobunyavirus*

## Abstract

**Background:**

Oropouche virus (OROV; *Orthobunyavirus*) is an emerging arbovirus endemic to South America and the Caribbean, with imported cases in European countries, including Spain. Although primarily transmitted by biting midges (*Culicoides* spp.), OROV has been detected in several mosquito species, raising concerns about potential establishment in nonendemic regions. European populations of *Aedes albopictus*, and *Culex pipiens*, as well as the invasive *Aedes aegypti*, represent relevant models for assessing vector competence.

**Objectives:**

Here, we evaluated the vector competence of Spanish *Cx. pipiens* biotype *molestus*, Spanish *Ae. albopictus*, and *Ae. aegypti* (Liverpool strain), for the 2024 OROV outbreak strain. Female mosquitoes were orally exposed to infectious blood meals and maintained under controlled insectary conditions at 27 °C. In addition, an additional group received a second noninfectious blood meal. The survival of the mosquitoes was monitored, and infection, dissemination, and transmission rates were assessed at 7, 14, and 21 days post infection. Vertical transmission of the virus to the progenies was also analyzed.

**Results:**

Overall, *Ae. albopictus* exhibited low infection rates, with occasional dissemination and transmission events. *Aedes aegypti* and *Cx. pipiens* biotype *molestus* showed infection and occasional dissemination, but no evidence of transmission. A second noninfectious blood meal did not significantly affect infection, dissemination, or transmission rates in any species. Viral loads in bodies and legs were low and did not differ significantly between species, time points, or feeding regimens. Survival was not affected by infection or blood-feeding regime. We did not find vertical transmission of OROV to the progenies.

**Conclusions:**

Regardless of virus dissemination in mosquitoes, our study indicates a poor vector competence of Spanish *Ae. albopictus* and no evidence of transmission under the conditions tested in *Ae. aegypti* and *Cx. pipiens* biotype *molestus* for the circulating OROV strain. These findings suggest a low risk for local OROV establishment in Spain, although continuous surveillance and research are warranted to monitor potential vector–virus adaptation.

**Graphical Abstract:**

**Figure:**
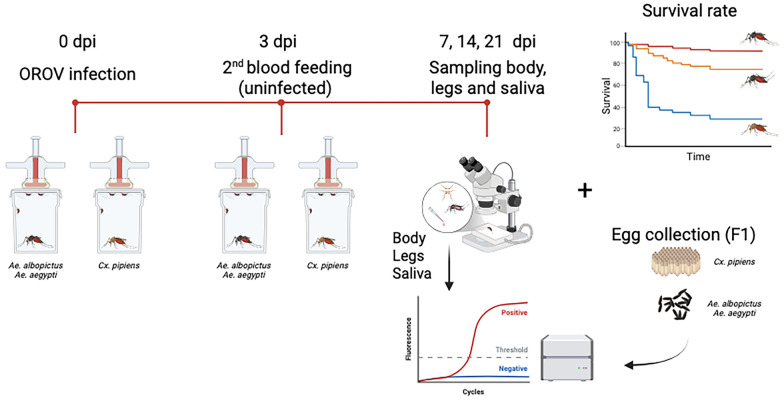

**Supplementary Information:**

The online version contains supplementary material available at 10.1186/s13071-026-07472-4.

## Background

Oropouche virus (OROV; *Orthobunyavirus*) is an emerging and neglected arbovirus endemic to South America and the Caribbean. However, in 2024, an epidemic of Oropouche fever resulted in more than 13,000 confirmed cases, including two deaths, reported in more than 11 countries in America [1]. This outbreak has renewed attention to OROV as an important public health concern. Although no autochthonous cases have been notified in Europe to date, a total of 30 imported cases have been identified in travelers returning from endemic regions, with Spain reporting the majority of these cases (*n* = 21) [2, 3].

Current evidence indicates that OROV is primarily transmitted by biting midges of the genus *Culicoides* (Ceratopogonidae), particularly *Culicoides paraensis*, which is considered the main vector in urban cycles [4]. However, OROV has also been detected in several mosquito species [5–12]. The virus was first isolated from a pool of *Aedes serratus* in Brazil in 1961 [12], and *Culex quinquefasciatus* has been proposed as a secondary, urban, anthropophilic vector after multiple isolations [4–6, 11]. Despite these findings, infection rates in mosquitoes have generally been low, suggesting limited susceptibility. Indeed, experimental oral infection studies have shown that *Cx. quinquefasciatus* is unlikely a competent vector of OROV [6, 8, 11].

The introduction of OROV into nonendemic regions through viremic travellers raises important questions regarding the competence of local *Culicoides* and mosquito populations to transmit the virus. There is increasing interest in Europe in assessing the vector competence of *Culicoides* and mosquito species, and consequently several research groups across Europe have recently published data on the vector competence of local species in their respective countries [13–15]. Spain has reported the highest number of OROV cases in Europe [3], highlighting the relevance of assessing the vector competence of mosquito species present in the country. In this context, both the invasive Asian Tiger mosquito, *Aedes albopictus*, and the autochthonous common mosquito, *Cx. pipiens*, are present throughout the country and could potentially be relevant models for OROV vector competence studies. Furthermore, *Ae. aegypti*, another potential model, while not present in mainland Spain, has been occasionally reported in the Canary Islands, Spain, in recent years [16, 17]*. Culex quinquefasciatus*, a member of the *Cx. pipiens* complex, is absent from Spain, but *Cx. pipiens* is widespread throughout Spain and could potentially act as a vector. In addition, In Europe, the *Culex pipiens* complex plays a key role in the transmission of several arboviruses, including West Nile virus [18]. This complex includes two main biotypes, *Cx. pipiens* biotype *pipiens* and *Cx. pipiens* biotype *molestus*, which differ in several ecological and behavioral traits. While biotype *pipiens* is typically associated with above-ground habitats and is considered predominantly ornithophilic, biotype *molestus* is commonly found in urban and underground environments and tends to feed more frequently on mammals, including humans. In addition, biotype *molestus* is capable of autogenous reproduction [19]. However, host-feeding patterns within the *Cx. pipiens* complex are flexible, and hybridization between biotypes occurs in natural populations, generating mosquitoes with intermediate host-feeding behavior [18, 19]. In Spain, the presence of *Cx. pipiens* biotype *molestus* has been widely reported, with estimated prevalence ranging from approximately 8.4% [20] to 60% [21] depending on the region.

The unprecedented scale of the 2024 OROV outbreak may be associated with a novel viral reassortant. This reassortant contains an M segment from strains detected in the eastern Amazon between 2009 and 2018, and L and S segments from strains circulating in Peru from 2008 to 2021 [22]. Such a reassortant replicates to higher titers in mammalian cells and shows reduced sensitivity to neutralization by human OROV immune sera collected before 2016 [23]. These genetic changes may influence viral pathogenicity and symptomatology [23]. Indeed, during the 2024 outbreak, OROV infection was linked to fatal cases in previously healthy young women as well as to fetal death and microcephaly [24]. Nevertheless, the consequences of viral genetic variation for the mosquito-mediated transmission of OROV remain poorly understood. Evidence from other arbovirus–vector systems suggests that even subtle genetic changes can have major effects on vector competence. For example, in the case of Chikungunya virus (CHIKV), a single amino acid substitution was shown to increase viral adaptation to *Aedes albopictus*, facilitating epidemic spread in areas where *Aedes aegypti* was absent [25]. Still, given the emergence of a novel reassortant strain, changes in viral pathogenicity or vector range cannot be excluded.

Moreover, increasing evidence shows that the acquisition of a second, noninfectious blood meal can significantly enhance arbovirus dissemination and shorten the extrinsic incubation period in mosquitoes [26–28]. This phenomenon is thought to result from transient microperforations in the basal lamina surrounding the midgut, facilitating viral escape into the hemocoel and dissemination throughout different tissues [26–28]. Such effects have been documented for several virus–vector systems [26–28] and may also apply to OROV.

Here, we evaluate the vector competence of *Cx. pipiens* biotype *molestus* and *Ae. albopictus* populations originally collected in Spain, as well as the *Ae. aegypti* Liverpool strain (LVP), for OROV. Additionally, we assess the capacity for vertical transmission of OROV and investigate whether a second noninfectious blood meal influences vector competence. This study aims to determine whether mosquito species present in Spain have the potential to support OROV transmission and thus represent an emerging risk for local virus establishment.

## Mthods

### Insects

An established colony of *Cx. pipiens* biotype *molestus* (originally collected from Arganda del Rey, Madrid, Spain, in 2024 and molecularly identified by polymerase chain reaction (PCR), targeting the CQ11 microsatellite region [29]) and an established colony of *Ae. albopictus* (collected in Barcelona, Spain in 2009) [30] were used during the assays. Additionally, an *Ae. aegypti* laboratory colony (LVP) was also included. All colonies were maintained at the National Center for Microbiology, Instituto de Salud Carlos III (Spain), under controlled insectary conditions (27 °C, 12:12 h light–dark photoperiod, 80 ± 5% RH) in an environmental chamber (Thermo Fisher Scientific, Waltham, MA, USA). Adults were provided with 30% sucrose solution ad libitum until infection. Mosquitoes were reared at the BSL-2 insectary and transferred to the BSL-3 insectary for vector competence studies.

### Virus and cells

The strain OROV SP2024 (GenBank ID: PQ329253) was used for experimental infections. This strain was originally isolated by the Laboratory of Arbovirus at the National Center for Microbiology, from a traveler returning from Cuba during the 2024 OROV outbreak [3]. Viral stocks were propagated once in Vero E6 cells (CRL 1586, American Type Culture Collection (ATCC), https://www.atcc.org), incubated at 37 °C for 4 days after which cytopathic effects were observed using a microscope, and freshly harvested supernatant was directly used for mosquito infections. The use of freshly propagated virus leads to higher infection and replication rates in mosquitoes compared with frozen aliquots, as has been demonstrated in other arboviruses, such as Zika virus (ZIKV) [31]. Viral supernatants were harvested at 96 h post infection and used immediately for mosquito oral infection assays. Each viral culture was initiated from the same previously titrated and aliquoted virus stock using identical inoculation doses and culture conditions to ensure comparable virus production across biological replicates. Viral titers were determined by tissue culture infectious dose 50 (TCID_50_) assay on Vero E6 cells, confirming equivalent infectious titers between replicates, following standard protocols to quantify infectious viral particles [32].

### Vector competence assay

Vector competence experiments were conducted with 3–7-day-old female mosquitoes. Approximately 100 females per species were placed in 450-ml cardboard cups covered with mesh custom-made by Alenta (https://www.alenta.org/) and were transferred to the BSL-3 insectary for mosquito infection and follow-up. Independent biological replicates were conducted for *Ae. albopictus* (*n* = 3), *Ae. aegypti* (*n* = 2), and *Cx. pipiens* biotype *molestus* (*n* = 5). Mosquitoes were deprived of sugar 24 h prior to feeding to stimulate blood ingestion. The infectious blood meal was prepared as a mixture of two parts of blood and one part of virus in a final volume of 3 ml and consisted of freshly collected defibrinated rabbit blood provided by a rabbit farm (San Bernardo Farm, Navarra, Spain) spiked with fresh OROV to a final titer of 2.5 × 10^7^ TCD_50_/ml, and supplemented with 5 mM ATP and 2.5% saturated sugar solution to stimulate blood-feeding. Mosquitoes were exposed to the infectious mixture through quail skin as a membrane at 37 °C for 45 min using a Hemotek feeding system (SP6W1-3; Hemotek Ltd, Blackburn, UK). After feeding, mosquitoes were anesthetized with CO_2_, and fully engorged females were separated on a chilled plate and transferred to new 450-ml cups and maintained with 30% sucrose ad libitum in an environmental chamber following the aforementioned rearing conditions at the BSL-3 insectary. Freshly engorged mosquitoes were collected at 0 days post infection (dpi) to confirm the acquisition of virus by reverse-transcription quantitative PCR (RT–qPCR), indicating successful virus uptake (viral titers average of 7.2 × 10^2^ TCID₅₀/sample in the body). For each species and experimental condition, between two and four mosquitoes were collected and processed individually. A subset of engorged females was offered a second noninfectious blood meal 3 dpi consisting of fresh rabbit blood following the same feeding protocol. Those females that successfully took the second blood meal were assigned to the “double-blood-meal feeding group” (from here, 2BF), whereas those that did not feed again were maintained as the “single-blood-meal feeding group” (from here, 1BF). The mosquitoes from both groups were introduced in 450-ml cardboard cups and provided with 30% sucrose solution ad libitum and kept in the environmental chamber (Climacell^®^, MMM Group, Germany) at 27 °C, 80% relative humidity (RH) and 12:12 light:dark cycle, in the BSL-3 insectary. Survival was monitored daily from Monday to Friday up to 21 dpi for the 1BF group and 14 dpi for the 2BF group.

At 7, 14, and 21 dpi, mosquitoes were anesthetized with CO₂, legs were removed, and individuals were induced to salivate. For this, cold-anesthetized mosquitoes were immobilized onto autoclave adhesive tape by sticking their wings and bodies to the tape. Their proboscis was introduced into 10 µL pipette tips containing 10 µL of minimum essential medium (MEM supplemented with 50 µg/mL penicillin–streptomycin, 50 µg/mL gentamicin, and 2.5 µg/ml fungizone) and salivation was induced by topically applying 1 µL of 2% pilocarpine (Novartis 2012, Alcon Cusí S.A. Barcelona, Spain) on the thorax. After 30 min, the MEM–saliva mixture retained in the pipette tip was added to a tube containing 300 μL of MEM supplemented with 50 µg/mL penicillin–streptomycin, 50 µg/mL gentamicin, and 2.5 µg/mL fungizone. Each mosquito body and corresponding legs and saliva samples were placed in separate microtubes containing 300 µL of the same medium and stored at −80 °C until analysis. Mosquito body and leg samples were manually homogenized with a sterile pellet pestle until complete tissue disruption was achieved, centrifuged at 6,000*g* for 5 min, and supernatants were used for RNA extraction.

To evaluate vertical transmission of OROV, after the infectious blood meal, 24 *Ae. albopictus*, 5 *Cx. pipiens* biotype *molestus,* and 26 *Ae. aegypti* engorged female mosquitoes were allowed to lay eggs (first gonotrophic cycle, F1) into plastic cups partially filled with water in which filter papers (for *Aedes* species) were placed. The eggs were allowed to hatch, and the larvae were reared in the environmental chamber in the BSL 3 and feed with TetraMin XL Flakes (Tetra, Germany) until adult emergence. For each species, five pools of five females (25 individuals) were tested for possible transmission of the virus to the F1 generation. The number of F1 individuals analyzed was determined by the number of successfully ovipositing females and the viable offspring obtained after the first gonotrophic cycle. Pooling strategies are commonly used in studies evaluating the vertical transmission of arboviruses in mosquitoes; however, the limited number of individuals analyzed may reduce the ability to detect rare transmission events.

All experimental infections and mosquito maintenance were conducted in a biosafety level 3 (BSL-3) insectary at the National Center for Microbiology, Instituto de Salud Carlos III (Spain), following institutional biosafety and biosecurity regulations. Additional data as Supplementary Material are available for exchange through the Viral Emergence Research Initiative platform (https://www.viralemergence.org/) (Additional file 1).

To determine vector competence status, mosquito bodies, legs and saliva were individually analyzed for the presence of OROV by specific RT–qPCR [33]. All samples with a quantification cycle (*Cq*) value < 38 were considered positive [3, 33]. Infection rate (IR), dissemination rate (DR), and transmission rate (TR) were calculated as following: IR = proportion of mosquitoes with a positive body among the total number of mosquitoes exposed to the infectious blood meal that had been analyzed; DR = proportion of mosquitoes with positive legs among mosquitoes with a positive body; and TR = proportion of mosquitoes with positive saliva among mosquitoes with positive legs. Viral load in mosquito samples (bodies, legs and saliva) were estimated using a standard curve generated from tenfold serial dilutions of a virus stock with known titer determined by TCID_50_ assay in Vero cells. Cq values obtained by RT–qPCR were converted into estimated viral titers by interpolation against the standard curve. Mean viral titers were calculated by averaging the estimated viral loads of positive individual mosquitoes within each experimental group. As expected, *Cq* values were inversely correlated with viral titers. To assess the presence of infectious virus, saliva samples positive for viral RNA (100 μL of MEM-saliva mixture) were further inoculated onto Vero cell monolayers and monitored for cytopathic effect (CPE). When no CPE was observed, up to three blind passages were performed to attempt virus isolation. Cells were monitored for CPE for 7 days before performing the next passage.

### Statistical analysis

Mosquito survival was analyzed using Kaplan–Meier survival analysis, and differences between individuals fed an infectious or a noninfectious blood-meal were assessed using log-rank (Mantel–Cox) tests. Data were grouped by species and feeding status (1BF, 2BF). Owing to the low number of control samples, survival rates of 2BF *Cx. pipiens* biotype *molestus* are not shown. Survival among the evaluated species for 1BF was also assessed.

To evaluate IR and DR, independent generalized linear mixed-effects models (GLMMs) with a binomial error distribution and logit link function were fitted. Mosquito species, blood-feeding groups (one or two blood meals), and days post infection (7, 14, and 21 dpi) were included as fixed factors, with the variable of the experimental biological replicate included as a random effect to account for variation among independent assays. Interaction terms between species and blood-feeding regime were tested to evaluate species-specific effects of the second blood meal on the IR. Because only one saliva sample tested positive, the statistical analysis for TR was not conducted owing to insufficient statistical power. Viral load data (log₁₀-transformed RNA concentration) from body and leg samples were analyzed using linear mixed-effects models (LMMs) with the same fixed and random structure. In all the models, residuals were inspected for normality and homoscedasticity. No overdispersion was observed in all the models developed, supporting an adequate model fit. All analyses were performed in R (v4.2.3; R Core Team, 2025) using the lme4 package. All plots were generated using GraphPad Prism version 10.0.3 (GraphPad Software, San Diego, CA, USA). Statistical differences were considered for *P* < 0.05.

## Results

A total of 1,044 *Aedes albopictus*, 295 *Aedes aegypti*, and 728 *Culex pipiens* females were exposed to an infectious blood meal. Feeding success differed markedly among species, with 228 (21.26%) *Ae. albopictus*, 250 (84.75%) *Ae. aegypti*, and 141 (19.37%) *Cx. pipiens* biotype *molestus* successfully engorged. Among engorged females, a second, noninfectious blood meal was taken by 54/134 (40.30%) *Ae. albopictus*, 35/122 (28.69%) *Ae. aegypti*, and 11/74 (14.86%) *Cx. pipiens* biotype *molestus* (Table 1).

**Table 1:** Number and percentage of blood-fed mosquitoes relative to the total number of exposed mosquitoes for each species and experimental replicate

Species	Replicate	1BF	2BF
*Ae. albopictus*	1	28/92 (30.45%)	–
	2	108/609 (17.73%)	29/87 (33.33%)
	3	89/343 (25.95%)	25/47 (53.19%)
*Cx. pipiens* biotype *molestus*	1	12/100 (12%)	–
	2	60/306 (19.61%)	9/54 (16.67%)
	3	5/97 (5.15%)	–
	4	55/173 (31.79%)	2/20 (10%)
	5	9/52 (17.31%)	–
*Ae. aegypti*	1	72/112 (64.29%)	14/29 (48.28%)
	2	178/183 (97.27%)	21/83 (25.30%)

Results are shown separately for a single infectious blood meal (1BF) and a secondary noninfectious blood meal administered after an initial infectious blood meal (2BF)

Mosquito survival was monitored to assess the impact of viral infection. Control groups fed exclusively on noninfectious blood meals included 82 *Ae. albopictus*, 61 *Ae. aegypti*, and 36 *Cx. pipiens* biotype *molestus* females. In addition, survival following a second blood-feeding was evaluated in *Ae. aegypti* (*n* = 23) and *Ae. albopictus* (*n* = 11) females exposed to two consecutive noninfectious blood meals.

**Fig. 1:**
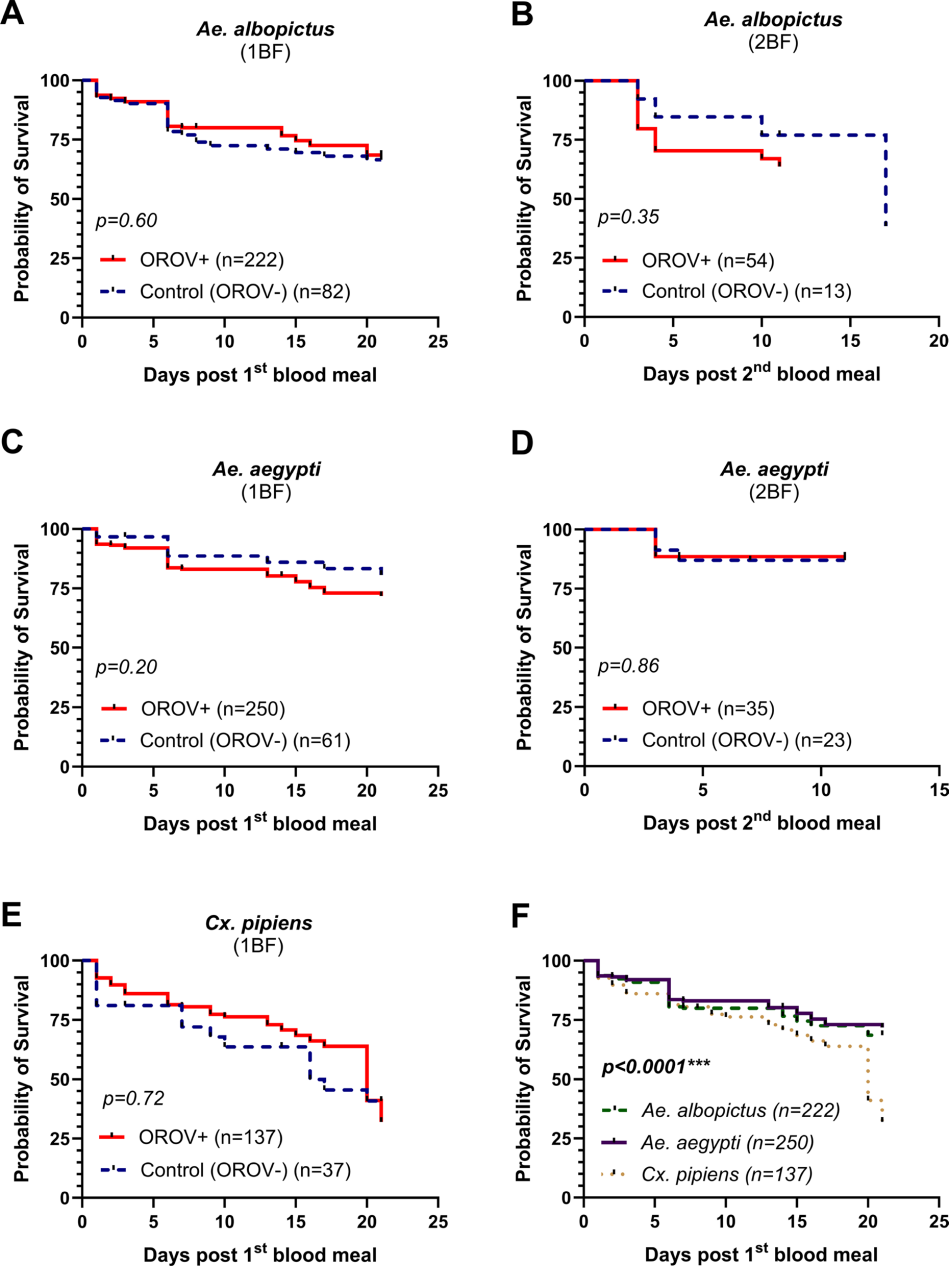
Survival of mosquitoes following Oropouche virus (OROV) exposure. Survival curves of *Aedes aegypti* (**A**, **B**), *Aedes albopictus* (**C**, **D**) and *Culex pipiens* biotype *molestus* (**E**) following exposure to infectious (OROV+) or noninfectious (control, OROV−) blood meal with one single infectious blood meal (**A**, **C**, **E**) or with an additional second noninfectious blood meal (**B**, **D**). **F** Comparative survival curves of the three mosquito species after OROV-infectious blood meal

No significant differences in survival were observed between infected and uninfected mosquitoes in any species (Fig. 1A, C, E). Moreover, a second noninfectious blood meal did not affect survival of mosquitoes that had previously ingested one blood meal (Fig. 1B, D). In contrast, survival differed significantly among species exposed to the infectious blood meal, with *Cx. pipiens* biotype *molestus* showing lower lifespan than *Aedes* species (Fig. 1F; *P* < 0.001).

**Fig. 2:**
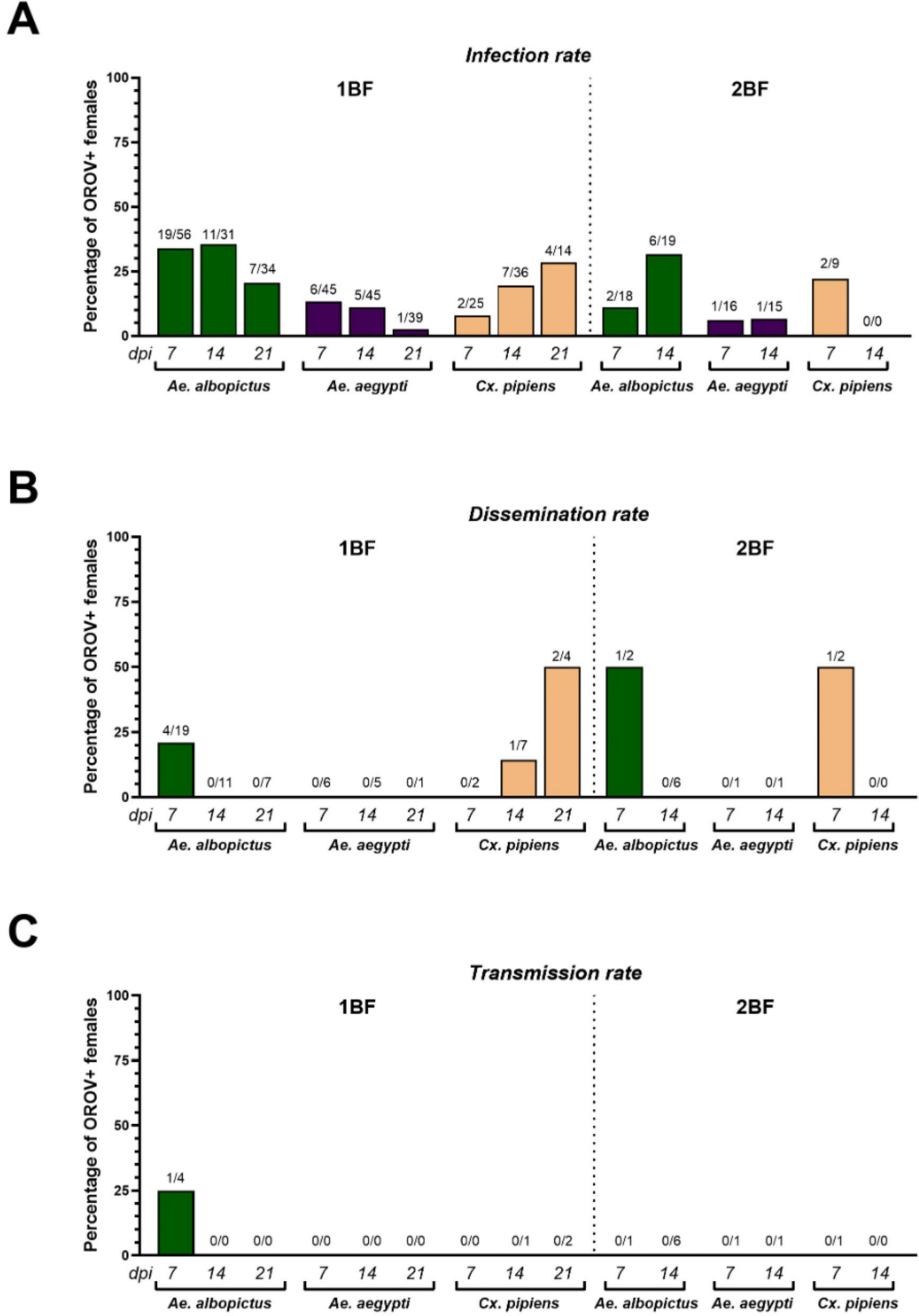
Infection, dissemination, and transmission rates of Oropouche virus (OROV) in *Aedes albopictus*, *Aedes aegypti*, and *Culex pipiens* biotype *molestus* following the first (1BF) and second blood-feeding (2BF). **A** Infection rates, **B** dissemination rates and **C** transmission rates observed in females analyzed at 7, 14 and 21 days post infection (dpi) after the first blood-feeding, and at 7 and 14 dpi after the second blood-feeding. Numbers above bars indicate the number of positive females over the total number tested

Vector competence was assessed in 159 *Ae. albopictus* (121 from group 1BF and 38 from group 2BF), 160 *Ae. aegypti* (129 from group 1BF and 31 from group 2BF), and 84 *Cx. pipiens* biotype *molestus* (75 from group 1BF and 9 from group 2BF). Full details on the number of mosquitoes tested per species and sampling time, IR, DR, TR, are presented in Fig. 2 and Table 2.

**Table 2:** Infection (IR; proportion of mosquitoes with a positive body among the total number of mosquitoes exposed to the infectious blood meal that had been analyzed), dissemination (DR; proportion of mosquitoes with positive legs among mosquitoes with a positive body) and transmission rates (TR; proportion of mosquitoes with positive saliva among mosquitoes with a positive legs) for the mosquito species experimentally infected with OROV

Species	DPI	BF	IR	DR	TR
*Ae. albopictus*	7	1	19/56 (33.93%)	4/19 (21.05%)	1/4 (25%)
		2	2/18 (11.11%)	1/2 (50%)	0/1 (0%)
*Ae. albopictus*	14	1	11/31 (35.48%)	0/11 (0%)	0%
		2	6/19 (31.58%)	0/6 (0%)	0%
*Ae. albopictus*	21	1	7/34 (20.59%)	0/7 (0%)	0%
		2	–	–	–
*Cx. pipiens* biotype *molestus*	7	1	2/25 (8%)	0/2 (0%)	0%
		2	2/9 (22.22%)	1/2 (50%)	0/1 (0%)
*Cx. pipiens* biotype *molestus*	14	1	7/36 (19.44%)	1/7 (14.29%)	0/1 (0%)
		2	–	–	–
*Cx. pipiens* biotype *molestus*	21	1	4/14 (28.57%)	2/4 (50%	0/2 (0%)
		2	–	–	–
*Ae. aegypti*	7	1	6/45 (13.33%)	0/6 (0%)	0%
		2	1/16 (6.25%)	0/1 (0%)	0%
*Ae. aegypti*	14	1	5/45 (11.11%)	0/5 (0%)	0%
		2	1/15 (6.66%)	0/1 (0%)	0%
*Ae. aegypti*	21	1	1/39 (2.56%)	0/1 (0%)	0%
		2	–	–	–

Results are shown separately for a single infectious blood meal (1BF) and a secondary noninfectious blood meal administered after an initial infectious blood meal (2BF)

DPI, days post infection; BF, blood-feeding group

**Fig. 3:**
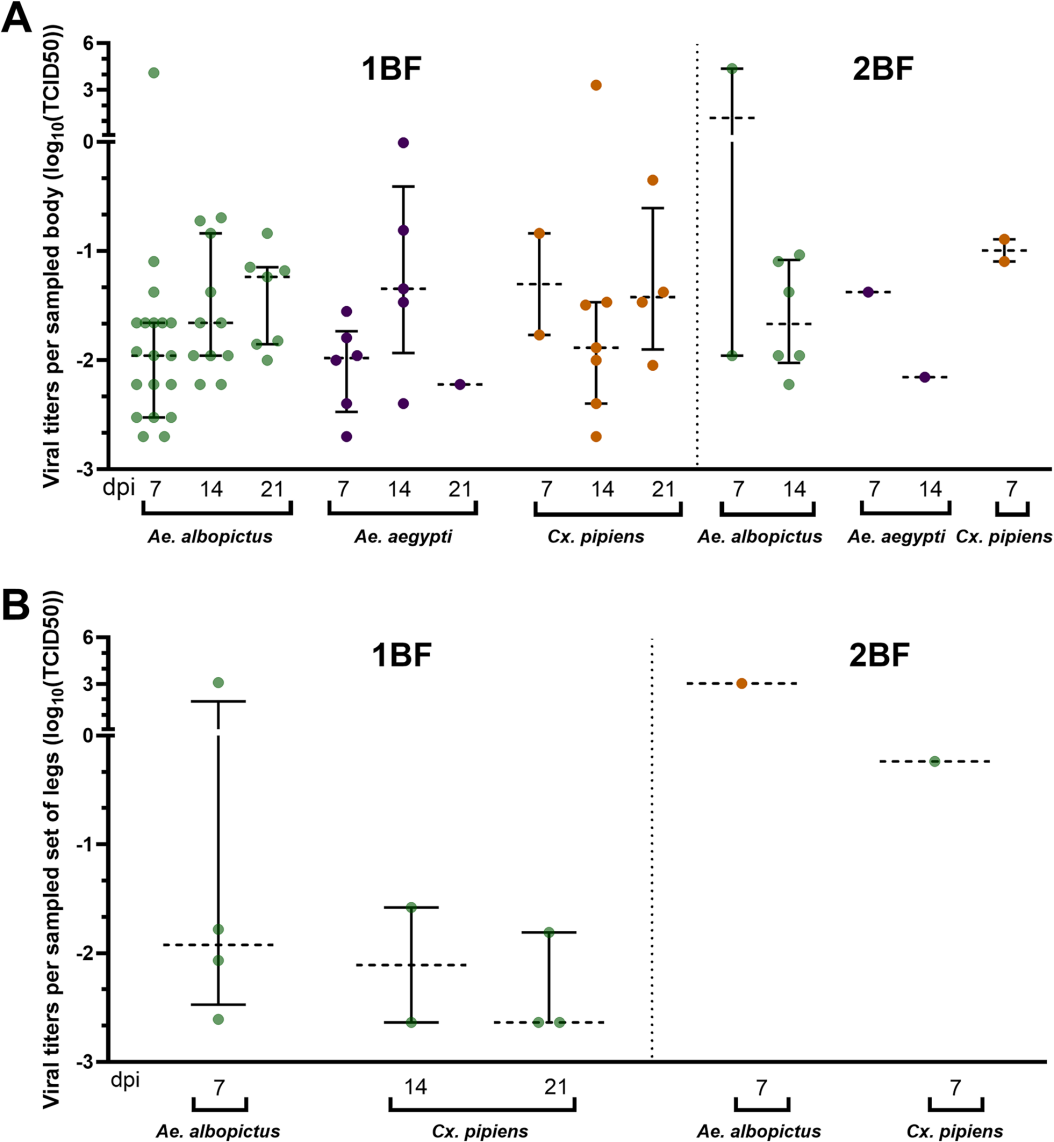
OROV viral titer per sample. Individual points represent obtained viral titer of bodies (**A**) and legs **B** of OROV-positive individuals. Data were grouped by mosquito species, day post infection (dpi), and feeding status (1BF, a single infectious blood meal; 2BF, a secondary noninfectious blood meal given to mosquitoes after an initial infectious blood meal). Median and interquartile values are shown for each study group

For mosquitoes of the 1BF group, *Ae. albopictus* exhibited IR of 33.93% at 7 dpi, 35.48% at 14 dpi, and 20.59% at 21 dpi. Dissemination occurred in 21.05% of infected individuals at 7 dpi, but it was absent at 14 and 21 dpi. Transmission was detected only once (1/4) at 7 dpi (Table 2, Fig. 2), corresponding to the individual that exhibited the highest viral load in both the body and legs (Fig. 3). Viral titer in this individual, calculated from RT–qPCR Ct values using a standard curve generated from serial dilutions of a virus stock with known titer, was 3.44 × 10^2^ TCID_50_/mL in the body, 3.2 × 10^2^ TCID_50_/mL in the legs, and 2.9 × 10⁻^3^ TCID_50_/mL in the saliva (Fig. 3). Despite the inoculation of one third of the saliva sample of that mosquito (100 μL) on Vero cells, no cytopathic effect (CPE) was observed after three passages. In the 2BF group, IR were 11.11% at 7 dpi and 31.58% at 14 dpi. Dissemination was detected in 50% of infected individuals at 7 dpi but not at 14 dpi, and no transmission was observed (Fig. 2).

For *Ae. aegypti*, in the 1BF group, IR were 13.33% at 7 dpi, 11.11% at 14 dpi, and 7.69% at 21 dpi. No dissemination or transmission was observed in *Ae. aegypti*. In the 2BF group, IR were 6.25% at 7 dpi and 6.67% at 14 dpi, with no events of dissemination or transmission (Table 2, Fig. 2)*.*

For *Cx. pipiens* biotype *molestus*, in the 1BF group, IR increased from 8% at 7 dpi to 19.44% at 14 dpi and 28.57% at 21 dpi. Dissemination was observed in 14.29% of infected mosquitoes at 14 dpi and 50% at 21 dpi, and transmission was never detected. In the 2BF group, 22.22% of mosquitoes became infected at 7 dpi, with dissemination in one of them (50%). Transmission was absent at all time points (Table 2, Fig. 2).

No significant effects of mosquito species, days post infection, or blood-feeding treatment were detected for infection rate, dissemination rate, or viral loads (all *P* > 0.05; see Supplementary Table S1 for full statistical results) (Additional file 2). Transmission was detected in only one *Ae. albopictus* mosquito, preventing any meaningful statistical analysis of TR across species, feeding groups, or days post infection.

Finally, across all three mosquito species, no viral RNA was detected in the F1 adult pools analyzed, suggesting that vertical transmission did not occur under the experimental conditions tested.

## Discussion

In this work, we evaluated the vector competence of the most common mosquito species present in Spain, including Spanish populations of *Ae. albopictus* and *Cx. pipiens* biotype *molestus,* for the emergent OROV strain responsible for the 2024 outbreak. Additionally, given the major role of *Ae. aegypti* as a global arbovirus vector and its recent detection in the Canary Islands [16, 17], *Ae. aegypti* (LVP) was also included in the assays. We also tested the effect of a second blood meal on vector competence, as well as the potential vertical transmission of OROV. Our results show that *Ae. aegypti* and *Cx. pipiens* biotype *molestus* are not competent vectors for this OROV strain, while *Ae. albopictus* exhibits limited susceptibility. Although OROV RNA was detected in the saliva of a single *Ae. albopictus* at 7 dpi, the low viral load, the absence of CPE in cell culture, and the unsuccessful virus isolation after three passages on Vero cells, suggest that this finding could reflect residual RNA rather than biologically meaningful transmission. However, given that only a fraction of the saliva sample was used for virus isolation, the possibility that small amounts of viable virus were present could not be completely excluded.

The epidemiological relevance of these findings is underscored by the widespread distribution of some of these mosquito species across Europe [17, 34], and particularly in Spain, which has reported the highest number of imported OROV cases in Europe to date [3], increasing the probability of virus–vector encounters in areas where competent or partially competent mosquito species are established. *Aedes albopictus* and *Cx. pipiens* complex dominate entomological surveys in urban areas of mainland Spain and the Balearic Islands [34, 35], while *Ae. aegypti* has been recently detected in the Canary Islands [17] and is established in other European regions, including Madeira [36] and parts of the Balkans [16]. Under these circumstances, experimental assessments of vector competence using local mosquito populations are essential to accurately evaluate the risk of autochthonous transmission and to inform surveillance and preparedness strategies.

Given the interest in Europe in evaluating the risk of autochthonous transmission of OROV, European groups have evaluated the vector competence of mosquito species such as *Ae. albopictus* or *Cx. pipiens* for OROV, reporting variable and sometimes contradictory outcomes, ranging from a complete absence of transmission to successful viral dissemination and, in several cases, transmission [13–15]. In Europe, recent studies using *Ae. albopictus* populations have generally reported low or limited infection and transmission rates for OROV. For example, Jansen et al*.* [14] observed low infection rates that varied with temperature and time post infection, with OROV strain TR 9760 (1955, Trinidad and Tobago) transmission detected only at 14 dpi in mosquitoes reared at 24 °C and at 21 dpi at 27 °C. Rosales-Rosa et al*.* [15] also documented low infection rates using the same strain (TRVL9760 strain; 1955, Trinidad and Tobago; accession numbers KC759122–24) and a strain isolated in 2024 from a patient of Cuba (OROV-IRCCS-SCDC_1/2024, imported from Cuba to Italy; GenBank accession numbers PP952117–19), with viral dissemination detected in infected mosquitoes but no virus detected in saliva. Similarly, Mancuso et al*.* [13] detected viral RNA in the bodies of a small number of individuals from an Italian *Ae. albopictus* population, but no dissemination or transmission was observed using also the strain OROV-IRCCS-SCDC_1/2024. Overall, these studies indicate that European *Ae. albopictus* populations display low susceptibility to OROV, with infection occurring at low rates and transmission being detected only under restricted experimental conditions.

The absence of OROV transmission observed in *Cx. pipiens* biotype *molestus* in our study is consistent with patterns reported in previous experimental studies. In *Cx. pipiens* populations from Italy, no infection, dissemination or transmission was detected [13]. Similarly, Rosales-Rosa et al. [15] reported that *Cx. pipiens* from Belgium was not susceptible and did not support viral replication leading to transmission. Although infection was detected in a *Cx. pipiens* biotype *pipiens* population from Germany, no virus was found in saliva [14]. To date, only a single study conducted in the USA has reported OROV transmission by *Cx. pipiens*, with viral RNA detected in the saliva of one individual using the strain OROV 240023 (GenBank accession number PQ064919.1, PQ064920.1, and PQ064921.1) [37]. Overall, OROV competence in *Cx. pipiens* appears to be limited and may vary geographically or depend on specific ecological or virological factors. These isolated observations highlight the need for further intraspecific assessments across diverse *Cx. pipiens* populations.

Despite being a globally important arbovirus vector, competent for dengue virus (DENV), CHIKV, and ZIKV [38], *Ae. aegypti* LVP strain, showed limited dissemination of OROV in our study. Similarly, previous studies also reported only transient infection and low IR, but no transmission, indicating that, *Ae. aegypti* is unlikely to support efficient OROV transmission [11, 39].

Overall, evidence gathered across multiple studies from different research groups, including our work, indicates that OROV shows a limited capacity to infect, disseminate and be transmitted by major European mosquito species, such as *Cx. pipiens* and *Ae. albopictus*. Nevertheless, contrasting results reported among European studies highlight the complexity of OROV-mosquito interactions and suggest that vector competence may be influenced by multiple factors, including viral strain, mosquito population genetics, temperature, and experimental design [40]. Temperature is a particularly important environmental determinant shaping vector competence, as it directly affects viral replication dynamics, dissemination efficiency, and the duration of the extrinsic incubation period. Recent experimental work evaluating OROV susceptibility across different mosquito species under multiple thermal regimes has shown that infection and transmission outcomes may vary considerably depending on the incubation temperature, found transmission only at 24 °C and 27 °C [14]. This result emphasizes the importance of environmental context when interpreting laboratory vector competence experiments. In our study, mosquitoes were maintained under a single controlled temperature, which may not capture the full range of thermal conditions experienced by mosquito populations in natural environments. Therefore, although our results indicate limited competence under the experimental conditions tested, they do not exclude the possibility that infection, dissemination, or transmission dynamics could differ under alternative temperature scenarios. Several previous studies have also relied on limited sample sizes or assessed vector competence at only one or two time points during the extrinsic incubation period. As a result, additional experimental studies are needed to better characterize the kinetics of OROV replication in mosquitoes, ideally incorporating multiple incubation temperatures, different viral strains, and larger experimental cohorts, to more accurately assess the potential for OROV emergence in Europe. In this context, methodological heterogeneity across studies, including differences in infectious dose, virus strain, and incubation temperature, likely contributes to the variability reported [40, 41]. These limitations underscore the need for more standardized experimental approaches [42] to facilitate robust comparisons and improve risk assessment of emerging arboviruses.

Our findings suggest that the principal barriers to OROV infection and transmission are likely located at the midgut level rather than resulting from intrinsic molecular incompatibilities between the virus and its mosquito hosts. Although viral dissemination to secondary tissues, as evidenced by virus detection in legs, was occasionally observed, these events were rare and occurred at very low frequencies. This pattern indicates that OROV infection is largely restricted at the level of midgut infection and/or midgut escape, thereby limiting subsequent viral dissemination and transmission [43].

Numerous studies have demonstrated that a second noninfectious blood meal significantly shortens the extrinsic incubation period of arboviruses such as DENV, CHIKV, or ZIKV in mosquitoes (e.g., *Ae. aegypti* and *Ae. albopictus*) by enhancing viral dissemination from the mosquito midgut, possibly through microperforations in the basal lamina [26, 27]. However, we did not observe significant differences in OROV dissemination between individuals receiving a second blood meal and those fed only once in any of the species analyzed, which agrees with recent published data where multiple blood meals did not affect OROV dissemination in *Ae. aegypti* [28]. Similarly, other studies, using the same *Ae. albopictus* strain than in our study, have reported no effect of a second noninfectious blood meal on vector competence for other arbovirus [44]. However, the small sample sizes, particularly the limited number of *Cx. pipiens* biotype *molestus* in the double-feeding group, may have reduced our statistical power to detect effects of secondary blood meals on OROV dissemination.

A limitation of our study relates to the relatively low feeding success observed for *Ae. albopictus* and *Cx. pipiens* biotype *molestus* under artificial membrane-feeding conditions. Such feeding rates are frequently reported in experimental infections involving these mosquito species and reflect the difficulty of reproducing natural host-feeding behavior under laboratory conditions [45]. Nevertheless, restricting analyses to successfully engorged females may introduce a potential selection bias if these individuals represent a subset of mosquitoes with higher physiological fitness. Although several independent biological replicates were conducted, and the final number of mosquitoes analyzed is comparable to those reported in other vector competence studies [13, 15], this potential bias should be considered when interpreting the results. In addition, several experimental groups, particularly those receiving a second blood meal, contained relatively small sample sizes, which may have reduced our statistical power to detect subtle effects on viral infection, dissemination or transmission. Finally, as with most vector competence experiments, our results were obtained under controlled laboratory conditions using a single viral strain and constant environmental parameters, which may not fully capture the ecological complexity influencing mosquito–virus interactions in natural settings. Future studies incorporating larger sample sizes, multiple viral strains, and environmental conditions will be valuable to further refine the assessment of OROV transmission risk in European mosquito populations.

The absence of virus particles in all F1 adult pools from the species analyzed suggests that vertical transmission did not occur under the experimental conditions tested, which is consistent with previous studies [13]. However, the relatively small number of individuals analyzed and the evaluation of a single gonotrophic cycle limit the strength of this conclusion. Vertical transmission of arboviruses in mosquitoes is often reported at low frequencies, and detecting such events may require larger sample sizes [46, 47]. Therefore, our results do not rule out the possibility of vertical transmission under different ecological or experimental conditions. Additional studies incorporating multiple gonotrophic cycles and larger samples sizes would be required to more robustly assess the potential for transovarial transmission of OROV in these mosquito species.

## Conclusions

Our results indicate limited vector competence of Spanish *Ae. albopictus* and no evidence of competence in Spanish *Cx. pipiens* biotype *molestus* and the *Ae. aegypti* LVP strain for the emergent OROV strain under the experimental conditions tested. Overall, these findings suggest a low potential for mosquito-borne OROV transmission under current conditions in Europe. Nevertheless, coevolutionary processes and shifts in vector–virus interactions that could facilitate OROV adaptation to new environments should not be overlooked. The recent detection of imported OROV fever cases in Spain and other European countries underscores the need for continuous vector surveillance and further studies considering additional mosquito populations, viral strains, and ecological conditions to better characterize the risk of OROV emergence, including the experimental evaluation of *Culicoides* populations.

## Supplementary Information

Additional file 1.

Additional file 2.

## Data Availability

Data supporting the main conclusions of this study are included in the manuscript.
